# T143. NOT A NUISANCE ANY MORE: GLOBAL FMRI SIGNAL AT REST, PROCESSING SPEED AND SYMPTOM SEVERITY IN SCHIZOPHRENIA

**DOI:** 10.1093/schbul/sby016.419

**Published:** 2018-04-01

**Authors:** Annabel Umeh, Peter Liddle, Susan Fancis, Lena Palaniyappan

**Affiliations:** 1 University College Cork; 2 University of Nottingham; 3 University of Western Ontario

## Abstract

**Background:**

Before data from resting-state functional magnetic resonance imaging (rs-fMRI) is analyzed, the global signal (GS) - average blood-oxygen level dependent (BOLD) signal across all voxels in the brain - is normally removed through global signal regression (GSR). This convention arose in order to control for changes in brain activity that are usually of no interest but may be caused by non-neuronal factors, including changes in respiratory rate, arterial CO2 levels or cardiac pulsation. However, recent studies have indicated that GS may systematically vary between patients with schizophrenia and healthy controls. In addition to testing this notion, we also studied if the dynamic variance of global signal across time carried any meaningful information that relates to overall symptom severity and information processing speed in patients with schizophrenia.

**Methods:**

Data was collected from 39 in a clinically stable, medicated early stage of schizophrenia (median duration of illness= 6.5 years), and 34 sex, age and parental socioeconomic-status matched healthy controls over a 10-minute period of eyes-open rest at TR=2.5s (3T Philips Achieva, 240 time points, dual-echo, gradient-echo EPI). Scores were obtained from the Signs and Symptoms of Psychotic Illness (SSPI) scale and the Digit Symbol Substitution Test (DSST). Rs-fMRI time-series data were motion-corrected (using 6 parameters), slice-time corrected, reoriented with structural images, band-pass filtered (0.01–0.1 Hz), scrubbed using ArtRepair for framewise displacement and transformed to MNI space. SPM8 and the advanced version of the Data Processing Assistant for resting-state fMRI (DPARSFA) were used for this purpose. GS mean was computed from all grey matter voxels using a template mask in MNI space, using grand mean scaling to a base of 1000, averaged across all time-points. The variance of GS across time (dynamic GS variance) was computed from the entire 10-minutes of acquisition (240 time points).

**Results:**

Independent-sample t-tests used to compare GS mean between controls (mean[sd] = 3135.1[1244.4]) and the SCZ group (mean[sd] = 3207.8[1191.7]) yielded no significant results [t(71) = -0.14, p = .89]. The temporal variance of GS did not differ between controls (mean[sd] = 113.05[59.41]) and the SCZ group (mean[sd] = 118.02[57.93]) [t(71) = -0.36, p = .72]. In the SCZ group there was a significant correlation between the total SSPI score reflecting overall illness severity (□ = -.322, p < 0.05) and the mean GS. This relationship was especially pronounced for the syndrome of Reality Distortion (rho = -.344, p < 0.05) and Disorganization (rho = -.303, p = 0.065), where higher symptom severity was seen in patients with lower mean GS.

Dynamic variance in GS was higher in healthy controls with lower mean DSST (r = -.364, p = 0.04), but no such relationship was seen in the SCZ group (r = .066, p = .694). Notably, when compared to controls (mean[sd] = 57.4[9.40]), patients (mean[sd]=42.4[9.97]) had significantly lower DSST scores [t(69) = 6.47, p < 0.001]. Neither GS nor GS variance related to root mean square of framewise displacement in the 2 groups.

**Discussion:**

We did not find an overall increase or reduction in the global signal in patients with schizophrenia. Nevertheless, the strength of global signal obtained from resting fMRI is related to severity of persisting symptoms of schizophrenia, whereas the dynamic variance of this signal relates to the speed of processing ability assessed outside the scanner in healthy subjects. With emerging evidence relating global signal to cognitive vigilance and overall brain connectivity, our results indicate that global signal is a parameter of interest that should not be automatically discarded in resting fMRI studies of schizophrenia

